# A 3D-printed platform for modular neuromuscular motor units

**DOI:** 10.1038/micronano.2017.15

**Published:** 2017-06-19

**Authors:** Caroline Cvetkovic, Max H. Rich, Ritu Raman, Hyunjoon Kong, Rashid Bashir

**Affiliations:** 1 Department of Bioengineering, University of Illinois at Urbana-Champaign, Urbana, IL 61801, USA; 2 Micro and Nanotechnology Laboratory, University of Illinois at Urbana-Champaign, Urbana, IL 61801, USA; 3 Department of Chemical and Biomolecular Engineering, University of Illinois at Urbana-Champaign, Urbana, IL 61801, USA; 4 Department of Mechanical Science and Engineering, University of Illinois at Urbana-Champaign, Urbana, IL 61801, USA

**Keywords:** 3D printing, neuromuscular junction, skeletal muscle, stereolithography, tissue engineering

## Abstract

A complex and functional living cellular system requires the interaction of one or more cell types to perform specific tasks, such as sensing, processing, or force production. Modular and flexible platforms for fabrication of such multi-cellular modules and their characterization have been lacking. Here, we present a modular cellular system, made up of multi-layered tissue rings containing integrated skeletal muscle and motor neurons (MNs) embedded in an extracellular matrix. The MNs were differentiated from mouse embryonic stem cells through the formation of embryoid bodies (EBs), which are spherical aggregations of cells grown in a suspension culture. The EBs were integrated into a tissue ring with skeletal muscle, which was differentiated in parallel, to create a co-culture amenable to both cell types. The multi-layered rings were then sequentially placed on a stationary three-dimensional-printed hydrogel structure resembling an anatomical muscle–tendon–bone organization. We demonstrate that the site-specific innervation of a group of muscle fibers in the multi-layered tissue rings allows for muscle contraction via chemical stimulation of MNs with glutamate, a major excitatory neurotransmitter in the mammalian nervous system, with the frequency of contraction increasing with glutamate concentration. The addition of tubocurarine chloride (a nicotinic receptor antagonist) halted the contractions, indicating that muscle contraction was MN induced. With a bio-fabricated system permitting controllable mechanical and geometric attributes in a range of length scales, our novel engineered cellular system can be utilized for easier integration of other modular “building blocks” in living cellular and biological machines.

## Introduction

Engineering living cellular machines requires the interaction of one or more cell types in an instructive environment^1^. These systems could be composed of micro- or macro-scale subunits engineered to cooperatively perform certain tasks. The modularity of these subunits (consisting of cells, tissues, and biomaterials, along with growth factors or other biochemical signals) allows for “forward-engineering” of the system by assembling the components in a diverse manner, like building blocks, thus expanding the functionality of the system. For example, we recently demonstrated a modular skeletal muscle tissue “ring” that could be coupled to a three-dimensional (3D)-printed skeleton to produce net motion^2^. This bio-integrated actuator (bio-bot) was a functional cellular system that exhibited dynamic and adaptive behavior based on both its inherent design and its surroundings.

Our previously demonstrated muscle-powered biological machines^2,3^ used externally applied electrical or optogenetic signals to stimulate an engineered skeletal muscle tissue to contract. Skeletal muscle is the principal actuator in many animals^4^, and its inherently modular and scalable nature renders it a natural component of many cellular systems. The ability to respond to stimuli by producing force (resulting in events such as fluid motion or net displacement, in a pump or motile bioactuator, for example) is an intuitive design principle of many systems. However, a more complex biological system with greater functionality would likely require the integration and coordination of multiple cell types, that is, moving from a homotypic cluster such as a cell sheet^5–7^ or an engineered muscle strip^8,9^ (with a singular cell type) towards a heterotypic co-culture such as a neuromuscular junction (NMJ), with multiple cell types^1^. *In vivo*, skeletal muscle fibers are innervated by the axons of somatic motor neurons (MNs) and do not inherently contract without stimulation from an excitatory neurotransmitter^10^.

Early research on the formation of NMJs in two dimensions (2D) has primarily focused on either the co-culture of excised or isolated muscle and neural tissues *in vitro*^11,12^, or the differentiation of mouse^13–16^ or human^17–19^ embryonic stem cells into MNs (usually through the formation of embryoid bodies or EBs), which were then co-cultured with excised or engineered muscle tissues. Beyond applications in regenerative medicine and therapeutics^20^, however, only a few studies have produced 3D NMJ platforms or applied neuromuscular research to living cellular systems using embryonic or neural stem cells^21–23^. Furthermore, there is a lack of research demonstrating the possibility of translating such an arrangement into a platform that is potentially autonomous, scalable, and forward-engineered, which are necessary characteristics of a mobile and functional cellular system or machine.

**Figure 1:**
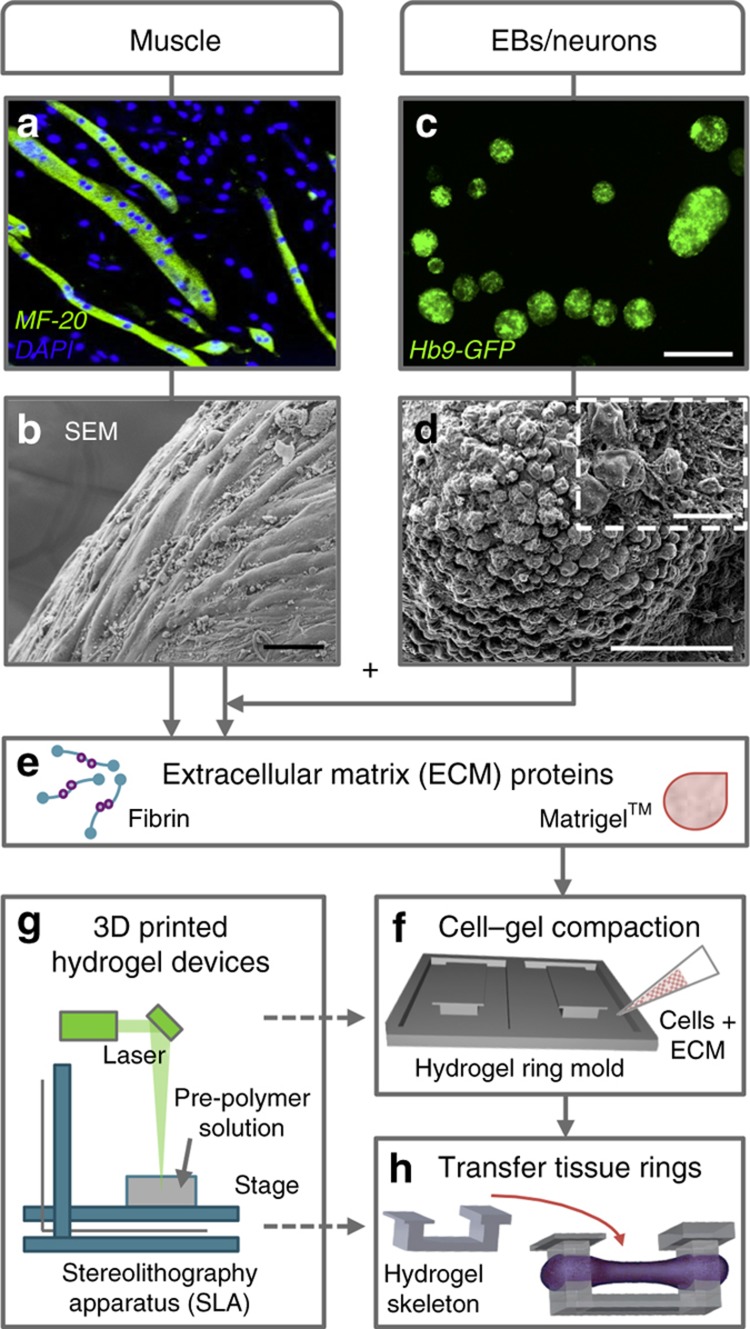
Skeletal muscle cells and motor neurons were combined into a fabricated 3D co-culture system. C2C12 myoblasts were differentiated into multinucleated myotubes (**a**) and combined with extracellular matrix (ECM) proteins to create an engineered muscle ring tissue (**b**). In parallel, mouse embryonic stem cells (HBG3 mESCs) were differentiated into motor neurons (MNs) through the formation of embryoid bodies (EBs) (**c** and **d**) and then combined with the engineered muscle tissue and ECM proteins (**e**) on 3D-printed hydrogel devices (**f** and **g**). Once the multi-layered rings sequentially compacted and fused together, they were then placed on a stationary hydrogel skeleton (**h**). Scale bars, 50 μm (**b** and **d**), 500 μm (**c**), and 10 Î¼m (**d**, inset).

Here we present a modular cellular system made up of multi-layered tissue rings containing integrated skeletal muscle and MNs embedded in an extracellular matrix (ECM). The first layer contained differentiated skeletal muscle myotubes (Figure 1a) mixed with ECM to form an engineered muscle tissue ring (Figure 1b). Simultaneously, MNs were differentiated from mouse embryonic stem cells (mESCs) through the formation of EBs, spherical aggregations of cells grown in suspension culture (Figures 1c and d). The EBs were mixed with ECM proteins (Figure 1e) to form a second tissue layer that integrated with the differentiated muscle tissue ring to create a co-culture amenable to both cell types. After the multi-layered rings sequentially compacted and fused together (Figure 1f), they were then placed on a stationary hydrogel skeleton that had been 3D printed in parallel (Figures 1g and h).

The use of stereolithographic 3D printing (an additive rapid prototyping technique)^24,25^ to create a flexible yet integrated tissue arrangement allows for iterative design modifications on a range of length scales. This system demonstrates functional NMJ behavior and controllable outputs, including engineered muscle contraction upon applied chemical stimulation, and permits control over physical, mechanical, and biochemical cues.

## Materials and methods

### Materials

3-(Trimethoxysilyl)propyl methacrylate, poly(ethylene glycol) diacrylate, retinoic acid, ciliary neurotrophic factor (CNTF), fibrinogen, thrombin from bovine plasma, aminocaproic acid (ACA), LONG R^3^ human insulin-like growth factor (IGF-1), Triton X-100, 4′, 6-diamindino-2-phenylindole (DAPI), and L-glutamic acid (glutamate) were obtained from Sigma-Aldrich (St Louis, MO, USA). Poly(ethylene glycol) dimethacrylate and hexamethyldisilazane (HMDS) were obtained from Polysciences, Inc. (Warrinton, PA, USA). 1-[4-(2-Hydroxyethoxy)phenyl]-2-hydroxy-2-methyl-1-propanone-1-one photoinitiator (Irgacure 2959) was obtained from BASF (Florham Park, NJ, USA). Dulbecco’s modified Eagle’s medium (DMEM), penicillin–streptomycin (10 000 U mL^−1^), and L-glutamine were obtained from Cellgro (Corning, Manassas, VA, USA). Fetal bovine serum (FBS) was obtained from VWR (Rednor, PA, USA). Mouse embryonic fibroblasts (CF-1 mitomycin-C inactivated MEFs) were obtained from Applied Stem Cell, Inc. (Milpitas, CA, USA). HBG3 mESCs (Hb9-GFP) were obtained from ArunA Biomedical (Athena, GA, USA). EmbryoMax ES DMEM, EmbryoMax nucleosides, ESGRO mouse leukemia inhibitory factor (mLIF), and purmorphamine were obtained from EMD Millipore (Billerica, MA, USA). Penicillin–streptomycin (5000 U mL^−1^), minimum essential medium (MEM) non-essential amino acids, β-mercaptoethanol, advanced DMEM/F12, neurobasal, KnockOut serum replacement, heat-inactivated horse serum, and collagen I-coated dishes were obtained from Gibco (Life Technologies, Carlsbad, CA, USA). Glial-derived neurotrophic factor (GDNF) was obtained from Neuromics (Edina, MN, USA). Matrigel basement membrane was obtained from Corning (Tewksbury, MA, USA). Paraformaldehyde was obtained from Electron Microscopy Services (Hatfield, PA, USA). Image-iT FX Signal Enhancer and tetramethylrhodamine α-bungarotoxin (TRITC-conjugated α-BTX) were obtained from Molecular Probes (ThermoFisher, Waltham, MA, USA). MF-20 anti-myosin heavy-chain antibody was obtained from the Developmental Studies Hybridoma Bank (The University of Iowa, Iowa City, IA, USA). Anti-glial fibrillary acidic protein (GFAP) was obtained from Chemicon (EMD Millipore). Alexa Fluor 488 goat anti-mouse IgG and Alexa Fluor 568 F(ab′)2 fragment of goat anti-mouse IgG were obtained from ThermoFisher. Thirty-five millimeter glass-bottom dishes used for imaging were obtained from MatTek (Ashland, MA, USA). (+)-Tubocurarine chloride hydrochloride pentahydrate (curare) was obtained from Abcam (Cambridge, MA, USA).

### Fabrication of hydrogel ring molds and bio-bot skeletons

CAD software (AutoCAD, Autodesk, San Rafael, CA, USA) was used to design hydrogel ring molds and bio-bot skeletons^2^. Briefly, parts were exported in.stl format, sliced into layers using 3D Lightyear software (v1.4, 3D Systems, Rock Hill, SC, USA), and fabricated using a modified Stereolithography apparatus (SLA 250/50, 3D Systems). Cover glass slides (22×22 mm^2^) were treated in an oxygen plasma system to render the surface hydrophilic, chemically treated with 2% (v/v) 3-TPM, and adhered to a 35-mm culture dish, as detailed previously^2^. This treatment ensured chemical tethering of the fabricated hydrogel to the underlying glass slide. For hydrogel ring molds and bio-bot skeletons, liquid pre-polymer solutions were prepared as previously described^3^: 20% (w/v) poly(ethylene glycol) dimethacrylate of *M*_W_ 1000 g mol^−1^ (PEGDMA 1000) and 20% (v/v) poly(ethylene glycol) diacrylate of *M*_W_ 700 g mol^−1^ (PEGDA 700), respectively, dissolved in phosphate-buffered saline (PBS) with 0.5% (w/v) Irgacure 2959. After fabrication, hydrogel parts were rinsed in PBS and then disinfected in 70% EtOH for at least 1 h. Sterilized parts were stored in sterile PBS at 4 °C until use.

### Cell culture

*Skeletal muscle*. Proliferating C2C12s (murine myoblasts) were maintained in a muscle growth medium consisting of DMEM with 10% (v/v) FBS, 1% (v/v) penicillin–streptomycin (10 000 U mL^−1^), and 1% (v/v) L-glutamine. Cells in culture were passaged before confluence. All cells, cultures, and tissue rings were incubated at 37 °C and 5% CO_2_.

*Mouse embryonic stem cells.* A feeder layer of mouse embryonic fibroblasts (CF-1 mitomycin-C-inactivated MEFs) was pre-plated 2 days prior to stem cell culture at a density of 3×10^4^ cells per cm^2^ in DMEM with 10% (v/v) FBS, 1% (v/v) penicillin–streptomycin, and 1% (v/v) L-glutamine. HBG3 mESCs (Hb9-GFP) were thawed and expanded at a density of 5×10^4^ cells per cm^2^ on top of the MEF layer in an mESC proliferation medium consisting of EmbryoMax ES DMEM with 15% (v/v) FBS, 1% (v/v) each of penicillin–streptomycin (5000 U mL^−1^), L-glutamine, EmbryoMax nucleosides and MEM non-essential amino acids, 0.1 mM β-mercaptoethanol, and 0.1% (v/v) mLIF. Cells were passaged before colonies reached confluence.

*EBs containing MNs*. As previously described^15,22^, to initiate differentiation, HBG3 mESCs were switched to neural differentiation medium containing 50% (v/v) advanced DMEM/F12, 50% (v/v) neurobasal, 10% (v/v) KnockOut serum replacement, 1% (v/v) penicillin–streptomycin (5000 U mL^−1^), 1% (v/v) L-glutamine, and 0.1 mM β-mercaptoethanol. After 1 h of incubation in serum-free neural differentiation medium, cells were trypsinized, centrifuged, and replated at a density of 1–2.5×10^6^ cells per 10-cm tissue culture dish (day 0). On day 1, floating cells in suspension were collected and replated in a new dish, and adhered cells were discarded. On day 2, floating EBs were collected and replated in a differentiation medium with 1 μM purmorphamine and 1 μM retinoic acid. On day 5, floating EBs were collected, and the medium was supplemented with 10 ng mL^−1^ each of GDNF and CNTF (a complete neural differentiation medium). Differentiated GFP^+^ EBs were used between days 5 and 7.

### Multi-layered tissue ring formation

For the formation of layer 1 of the multi-layered tissue rings, 5×10^6^ cells per mL (final density of C2C12s in the cell–gel solution) were combined with fibrinogen (4 mg mL^−1^), thrombin (0.5 U mg^−1^ fibrinogen), and Matrigel basement membrane (30% (v/v) of total cell–gel solution) on ice. Muscle growth medium was added to bring the cell–gel solution to its final volume (80 μL, 4×10^5^ cells total per ring, unless otherwise noted), and the solution was added to the well of a hydrogel ring mold (day 0). Molds were previously aspirated of excess liquid to ensure consistent cell–gel densities. Tissue rings were allowed to incubate for 2 h before adding warm growth medium with 1 mg mL^−1^ ACA, which was exchanged daily. On day 1, tissue rings were switched to a muscle differentiation medium consisting of DMEM with 10% (v/v) heat-inactivated horse serum, 1% (v/v) penicillin–streptomycin, 1% (v/v) L-glutamine, 1 mg mL^−1^ ACA, and 50 ng mL^−1^ IGF-1. On day 3, layer 2 was added to the hydrogel ring molds and allowed to compact around layer 1. Differentiated EBs were individually selected for being GFP^+^ and mixed with the cell–gel solution. The cell–gel solution (60 μL, 3×10^5^ cells total per ring) was otherwise identical to layer 1. Tissue rings were allowed to incubate for 2 h before adding a warm complete neural differentiation medium with 1 mg mL^−1^ ACA and 50 ng mL^−1^ IGF-1.

### Imaging

*Immunocytochemistry*. Samples were rinsed in PBS, fixed in 4% paraformaldehyde for 30 min, rinsed again, permeabilized with 0.25% Triton X-100 for 10 min, and blocked in Image-iT FX overnight at 4 °C. Samples were incubated with primary antibodies, MF-20 (1:400) or GFAP (1:1000), in Image-iT FX at 4 °C overnight. After rinsing three times with PBS, samples were incubated with secondary antibodies, either Alexa Fluor 488 or Alexa Fluor 568 goat anti-mouse (1:400), in Image-iT FX in the dark at 4 °C overnight. After rinsing three times with PBS, samples were incubated with DAPI (1:5000 in sterilized de-ionized water) for 10 min, rinsed, and imaged in a 35-mm glass-bottom dish using a confocal microscope (LSM710, Zeiss, Oberkochen, Germany). For imaging of acetylcholine receptors (AChRs), live samples were first incubated with TRITC-conjugated α-BTX (1:1000) in a complete neural differentiation medium for 1 h at 37 °C, and then fixed and imaged as detailed above.

*Scanning electron microscopy*. Samples were rinsed in PBS, fixed in 4% paraformaldehyde, and dehydrated using a series of ethanol solutions: 37% (10 min), 67% (10 min), 95% (10 min), and 100% (3×10 min). HMDS was added for 5 min and then allowed to vaporize. Gold/palladium was deposited on the dried sample for 70 s using a sputter coater (Desk II TSC, Denton Vacuum, Moorestown, NJ, USA). Images were acquired using an environmental scanning electron microscope (XL30, Philips/FEI, Eindhoven, The Netherlands).

*Area measurements*. A digital camera (Flex, SPOT Imaging Solutions, Diagnostic Instruments, Inc., Sterling Heights, MI, USA) on a stereomicroscope (MZ FL III, Leica Microsystems, Wetzlar, Germany) was used to take images of the multi-layered tissue rings during compaction. SPOT Software (v5.2, SPOT Imaging Solutions, Diagnostic Instruments, Inc., Sterling Heights, MI, USA) and ImageJ software (National Institutes of Health, Bethesda, MD, USA) were used to measure tissue area dimensions over time.

*Neurite growth measurements*. After day 9 of differentiation, EBs were plated on either collagen I- or Matrigel-coated dishes in a complete neural differentiation medium. EBs containing MNs expressed GFP^+^ and thus did not require additional cell markers. Live samples were imaged on days 10–14 using an inverted fluorescent microscope for 2D cultures (IX81, Olympus, Tokyo, Japan) or a confocal microscope for 3D tissue rings (LSM710, Zeiss, Oberkochen, Germany). The NeuronJ plug-in for ImageJ (National Institutes of Health, Bethesda, MD, USA) was used to measure neurite growth distances (Supplementary Figure S1).

### Chemical stimulation of multi-layered tissue rings

To stimulate MNs in tissue rings, glutamate was added to the cell culture medium in a bath application of either 200 or 400 μM, as noted. Tissues were then transferred to the fresh medium. After chemical stimulation, the nicotinic AChR antagonist curare was added to the cell culture medium in a bath application of 25 μM. Tissues were then rinsed in PBS and transferred to fresh medium.

### Video capture and movement tracking

Muscle contraction within tissue rings was captured using a digital camera on a stereomicroscope with a capture rate of 5–10 frames per second. Image sequences were exported to .avi files. A custom MATLAB script was used to calculate the *x*–*y* displacement of user-specified regions of interest using normalized 2D cross-correlation, as described previously^3^.

### Statistical analysis

All results are presented as the mean±standard deviation. OriginPro software (v9.1, OriginLab, Northampton, MA, USA) was used to calculate significance (one-way analysis of variance followed by Tukey’s multiple comparison test).

## Results

### Differentiation of EBs containing MNs

**Figure 2:**
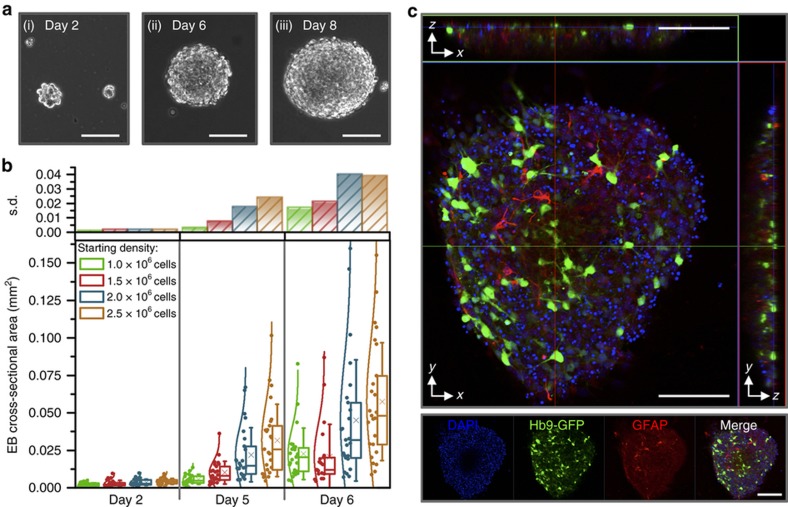
(**a**) EBs aggregated more cells, grew in diameter, and became more spherical over time (shown at (i) day 2, (ii) day 6, and (iii) day 8 of differentiation). (**b**) Embryoid bodies (EBs) increased in size over a week of differentiation. The starting cell density of HBG3 mESCs influenced the size of mature EBs, with a larger initial density resulting in larger average cross-sectional area, as well as a larger range (standard deviation, s.d.) of diameters of resulting EBs. Box plots represent 25th, 50th, and 75th percentiles, with average values marked as (*x*) and whiskers representing±s.d. Data are presented (along with normal distribution curves) to the left of the boxes (*n*=20–25 EBs per density and time point). (**c**) Confocal images (*xy*, large box; *xz*; *yz*) of an EB (day 8 of differentiation) demonstrated the presence of Hb9-GFP^+^ motor neurons (green), as well as glia (red), throughout the entire EB. All scale bars represent 100 μm.

To attain MNs, we induced mESCs (HBG3 mESCs, from a transgenic mouse cell line) to directly differentiate using a protocol that recapitulates spinal MN maturation in embryonic development *in vivo*^13,15,22^. Temporal addition of relevant growth and signaling factors pushed cells to become neural progenitor cells and then MNs. First, HBG3 mESCs were proliferated on a feeder layer of MEFs to support their propagation, preserve pluripotent capacity, and prevent differentiation^26^. When colonies became confluent (Supplementary Figure S2a), cells were switched to a differentiation medium, trypsinized, centrifuged, and replated in a cell culture dish. MEFs adhered to the dish, while HBG3 mESCs differentiated in suspension and aggregated to become spherical EBs (Figure 2a). We added retinoic acid and purmorphamine (caudalizing and ventralizing signaling molecules that drive neural progenitors first toward spinal and then MN identities, respectively^13^) on day 2 and supplemented EB cultures with GDNF and CNTF (neural growth factors that promote MN survival^27^) on day 5. The EBs increased in size and circularity over time, with the initial cell seeding density on day 0 playing a role in the size of differentiated EBs (Figure 2b).

Cells expressed green fluorescent protein (GFP) under the control of the post-mitotic MN-specific Hb9 promoter (Supplementary Figure S3a); thus, we could visually confirm differentiation of MNs without the addition of exogenous factors or antibodies. Fluorescent imaging revealed that EBs also contained proliferating glia (Figure 2c), which are known to contribute to the formation and maintenance of the NMJ and differentiation and survival of MNs^11^.

**Figure 3:**
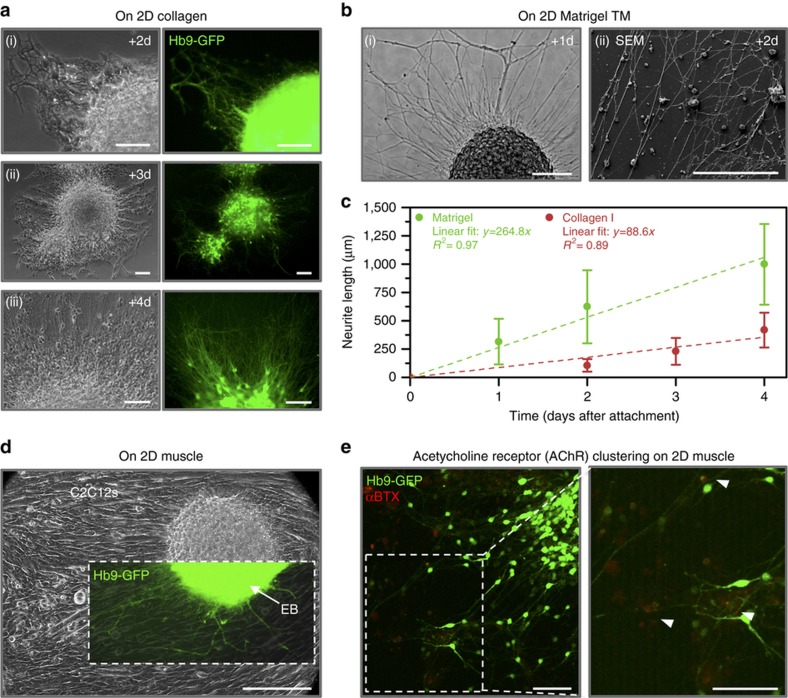
Differentiated embryoid bodies attached readily to 2D ECM-coated substrates such as (**a**) collagen I (shown after (i) 2, (ii) 3, and (iii) 4 days of attachment) and (**b**) Matrigel (shown after (i) 1 and (ii) 2 days of attachment). All scale bars represent 100 μm. (**c**) The EBs extended GFP^+^ neurites from motor neurons across the surface of the gels upon attachment. The plot represents the mean±standard deviation (*n*=103–298 neurites from three to seven images per time point). (**d**) EBs also attached to 2D cultures of differentiating C2C12s and extended neurites across the surface of the myotubes (shown after 1 day of attachment). The scale bar represents 200 μm. (**e**) Clusters of post-synaptic acetylcholine receptors (AChRs, red, stained with α-bungarotoxin) were visible near the termination of neurite extensions on myotubes 5 days after co-culture. The scale bars represent 100 μm.

When allowed to adhere to natural ECM substrates such as collagen I (Figure 3a) and Matrigel (Figure 3b and Supplementary Figure S3b), EBs containing MNs attached readily and extended neurites across the gels. Neurites grew 417.5±154.8 and 999.1+356.9 μm after 4 days of attachment (propagating at nearly linear rates of 88.6 and 264.8 μm per day) on collagen I and Matrigel surfaces, respectively (Figure 3c). Similarly, EBs adhered to (and extended neurites across) the surface of 2D layers of differentiated C2C12s (Figure 3d and Supplementary Figure S3c). After 5 days of co-culture, we identified clusters of post-synaptic AChRs (visualized with fluorescently conjugated α-BTX, which binds to a subunit of AChR) near the termination of neurite extensions on myotubes (Figure 3e).

### Development of multi-layered tissue rings in hydrogel molds

**Figure 4:**
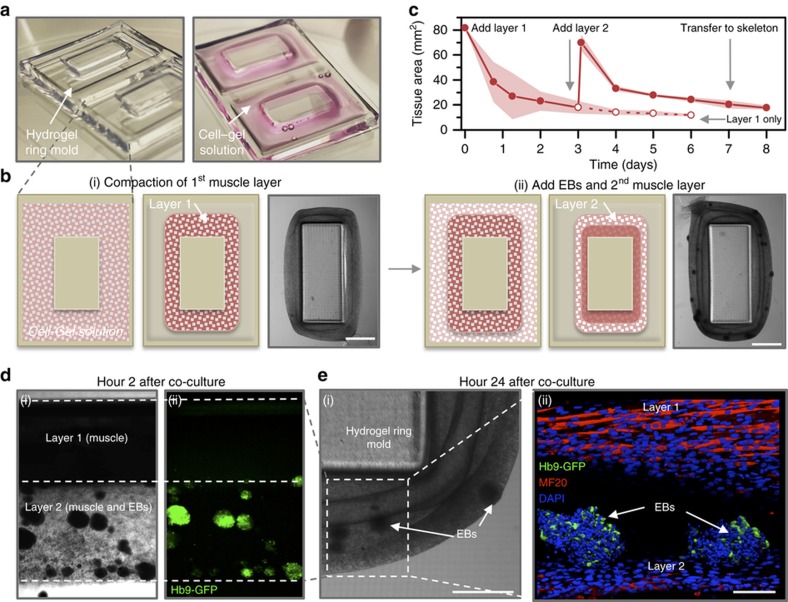
(**a**) A 3D-printed hydrogel structure was used as a mold to guide the formation of a liquid cell–gel solution into a solid engineered muscle tissue. (**b**) Schematic of the two-layer muscle–neuron ring system. (i) The first layer, consisting of myoblasts and ECM proteins, compacted to form a solid muscle ring. (ii) After 3 days, the cell–gel solution containing differentiated EBs was added to the mold and compacted around (and fused to) the first layer to form a second layer. Both images are shown 48 h after cell seeding for each layer. The scale bars represent 2 mm. (**c**) As the cell–gel solution compacted to form a muscle ring, the cross-sectional tissue area decreased over time. The plot represents the mean±standard deviation (shaded area; *n*=3–6 rings per time point). (**d**) Compaction of layer 2 brought the EBs in close contact with differentiated muscle in layer 1. (i) Phase-contrast and (ii) fluorescent images demonstrated the presence of EBs in layer 2, 2 h after co-culture. The scale bar represents 1 mm. (**e**) Phase (i) and confocal images (ii) of the two-layer system 24 h after co-culture demonstrated multinucleated myotubes in layer 1 and GFP^+^ motor neurons in the EBs in layer 2. The scale bar represents (i) 1 mm and (ii) 100 μm.

To fabricate the macroscopic structures necessary for the development and co-culture of engineered tissue rings, we used a stereolithography apparatus to 3D print poly(ethylene) glycol-(PEG)-based hydrogels. We designed and 3D printed a hydrogel ring mold to guide the formation of a liquid cell–gel solution into a solid engineered muscle tissue. The mold contained rectangular-shaped wells that forced the compacting cells and ECM into a ring-shaped tissue (Figure 4a and Supplementary Figure S4a). A cell–gel solution consisting of 80 μL of C2C12 myoblast precursor skeletal muscle cells and ECM proteins (Matrigel and fibrinogen) was injected into the mold. The polymerization of the matrix proteins (thrombin was added to cleave fibrinogen and form a cross-linked fibrin network) and the traction forces exerted by the myoblasts on surrounding proteins resulted in compaction of the tissue into a solid ring (Figures 4b–i). After 1 day, we added a differentiation medium containing 10% horse serum to induce fusion of myoblasts into mature muscle fibers, or myotubes (Supplementary Figure S2b). The medium was also supplemented with ACA, an inhibitor to prevent degradation of the ECM by cell-secreted proteases) and insulin-like growth factor (IGF-1, which is known to increase myoblast fusion and muscle hypertrophy^28,29^).

After 3 days of allowing the first muscle layer to compact and differentiate in the hydrogel ring mold, we added a second layer of cell–gel solution (Figure 4bii). This mixture contained the same C2C12 and ECM components as the first layer, as well as differentiated EBs that were individually selected for being predominantly GFP^+^ (thus containing MNs). The co-culture medium was switched to a complete neural differentiation medium supplemented with ACA and IGF-1.

Layer 2 compacted around the first, and the cross-sectional tissue area decreased daily to a lower limit of 17.9±1.6 mm^2^, or ~22% of the original area dictated by the hydrogel ring mold, after 5 days of co-culture (Figure 4c). We controlled the thickness of the tissue by varying the initial cell–gel volume; initial volumes of 60, 80, and 90 μL (containing 3×10^5^, 4×10^5^, and 4.5×10^5^ cells in each ring) resulted in ring tissues with layer 1 cross-sectional areas of 11.7±1.6, 13.9±2.4, and 17.1±4.6 mm^2^ (14.3%, 17.1%, and 21.0% of the original area) after 5 days, respectively (Supplementary Figure S5).

Various imaging modalities confirmed the presence of both differentiated muscle and GFP^+^ EBs in the multi-layered tissue rings. Compaction of layer 2 brought the EBs in close contact with layer 1 during the first 24 h of co-culture (Figure 4d). By this time, the myoblasts in layer 1 had differentiated to form elongated, multinucleated myotubes that expressed mature myosin protein (Figure 4e and Supplementary Video S1).

### Spontaneous muscle contraction in co-culture

**Figure 5:**
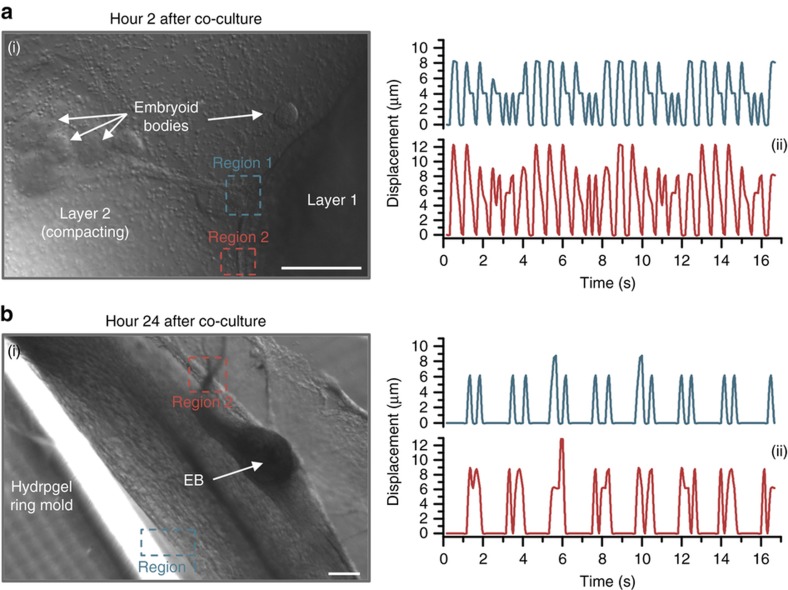
Spontaneous contraction of the muscle in layer 1 was observed at (**a**) 2 and (**b**) 24 h after the addition of layer 2 containing EBs to the hydrogel ring molds. (i) Phase-contrast images demonstrating two regions where spontaneous contraction was observed. (ii) Displacement was measured for two regions of muscle contraction, which followed a periodic pattern and varied slightly depending on proximity to EBs. The frequency of contraction decreased between hours 2 and 24. All scale bars represent 200 μm.

Following the addition of layer 2 with MN-containing EBs to the hydrogel ring molds (Figure 5ai), we observed spontaneous contraction of differentiated muscle in layer 1 as early as 2 h after co-culture. Two regions of muscle in layer 1, ~600 and 800 μm from the nearest group of EBs, twitched at ~1.6 contractions per second, with local displacements measuring 9.9±1.8 and 6.8±1.9 μm per twitch for regions 1 and 2, respectively (Figure 5aii). After 24 h of co-culture, after layer 2 had begun to compact into a solid ring (Figure 5bi), we observed continued spontaneous contraction. The muscle twitched with decreased frequency (~0.9 contractions per second) and amplitude (6.5±0.9 and 8.7±1.6 μm per twitch) compared to hour 2 (Figure 5bii and Supplementary Video S2), and spontaneous contraction eventually ceased.

### Transfer of multi-layered tissue rings to hydrogel skeletons

**Figure 6:**
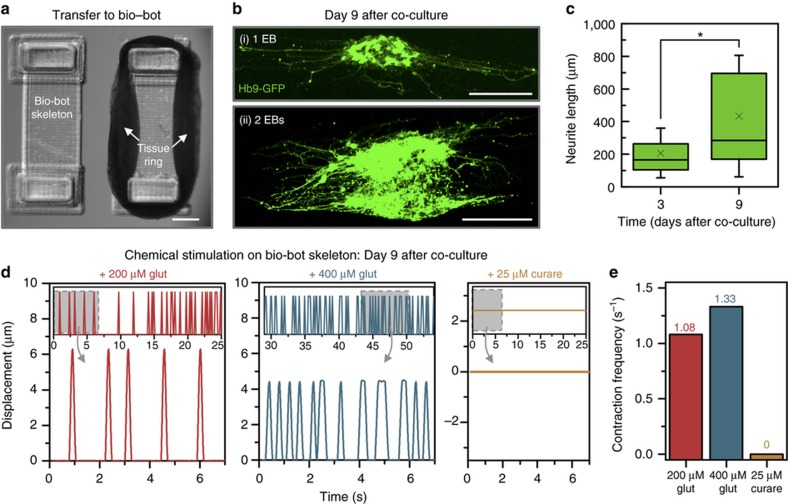
(**a**) Muscle–neuron tissue rings were transferred to 3D-printed bio-bot skeletons, made of a beam (chemically tethered to an underlying glass slide) connecting two pillars. Top view: the scale bar represents 1 mm. (**b**) Soon after the addition of layer 2, (i) single or (ii) groups of EBs began to extend neurites outward through the tissue (shown here 9 days after co-culture). The scale bars represent 200 μm. (**c**) Neurite growth from EBs in tissue rings significantly increased over time. Box plots represent 25th, 50th, and 75th percentiles, with average values marked as (*x*) and whiskers representing±standard deviation (*n*=51–185 neurites from 4 to 10 EBs per time point). (**d**) Video recordings demonstrated that the site-specific innervation of a group of muscle fibers allowed for muscle contraction via chemical stimulation, with the frequency of contraction increasing with glutamate concentration. The addition of 25 μM curare halted the contractions, indicating that muscle contraction was motor neuron-induced. (**e**) The frequency of contraction increased with glutamate concentration (from 1.08 to 1.33 contractions per second with 200 and 400 μM glutamate, respectively). After the addition of 25 μM curare, the frequency decreased to 0 observed contractions. The plot represents the mean values.

When both layers fused into one tissue, the compliant ring was physically placed onto a 3D-printed structure (Figure 6a). We designed and fabricated a stationary skeleton, composed of a pillar connecting two stiff beams, representing a physiological muscle–tendon–bone arrangement (Supplementary Figure S4b). The beam of the skeleton was chemically tethered to a glass slide and thus provided a static mechanical stretch to the tissue rings.

Within the multi-layered tissue rings, EBs began to extend neurites from GFP^+^ MNs. Using confocal imaging, we confirmed that the extension propagated in 3D throughout the tissue, both in the direction of other EBs and toward differentiated muscle (Figure 6b and Supplementary Video S3). Neurite length significantly increased between days 3 and 9 of co-culture, from 207.4±153.2 to 433.6±372.5 μm (Figure 6c).

### Chemical stimulation of MNs in multi-layered tissue rings

On day 9 after co-culture, we chemically stimulated MNs by adding glutamate. Multi-layered tissue rings were first subjected to a bath application of 200 μM glutamate in a complete neural differentiation medium. Through video recordings, we observed that local muscle contraction began in response to chemical stimulation of MNs (Figure 6d). When we increased the glutamate concentration from 200 to 400 μM, the frequency of muscle twitching increased from 1.08 to 1.33 contractions per second, whereas the average displacement decreased from 6.3±0 to 4.4±0.2 μm per twitch (Figure 6e and Supplementary Video S4). The addition of 25 μM tubocurarine chloride (curare), an irreversible nicotinic NMJ antagonist and muscle relaxant^23^ that blocks AChRs, halted the contractions; no further muscle twitching was observed.

## Discussion

The engineered hydrogel-muscle ring platform, which we demonstrated previously for muscle-powered biological machines^2^, is ideal for introducing different cell types and biomaterials. Here we present a method for overcoming some of the challenges associated with innervating 3D muscles^15^. Prior work has confirmed that ESC-derived MNs attained electrophysiological properties that were characteristic of native spinal MNs^14^; we demonstrate an ability to integrate MN-containing EBs into a cellular system and achieve outputs representative of a functional NMJ. A ring tissue design with directional force production allowed for a physiological neuron-muscle co-culture with greater potential for innervation in 3D, whereas an adaptable fabrication system provided physical cues and structural support for maturation and synergy of both neurons and muscle in a relevant engineered tissue system. By allowing the two major cell types to differentiate in parallel before combining them into one co-culture system, we were able to create a flexible platform in which cells and tissues can be combined with 3D-printed scaffolds in a modular and user-friendly manner.

Compaction in the hydrogel ring mold or transfer to the skeleton did not hinder the further maturation of either major cell type. C2C12s differentiated into mature myotubes in the presence of IGF-1, whose use we previously reported to accelerate muscle differentiation in 3D engineered systems in a physiologically relevant manner^3^. We also hypothesized that forcing the tissue to compact and differentiate in this constrained environment would result in greater myotube alignment along the longitudinal axis^2^, as the imposition of this static mechanical cue during muscle development would contribute to improved functionality and force production^30,31^. The design and fabrication of an instructive environment for this cellular system were easily achieved with the use of stereolithographic 3D printing^24,25^. This manufacturing technology has been widely utilized for applications in tissue engineering, not only due to the user’s control over the specific design, geometric, and mechanical parameters but also for its ability to fabricate biomaterials (hydrogels whose properties can mimic cells’ natural micro-environments) and encapsulate various cell types in three dimensions^32,33^ in a short time frame and over a range of length scales.

The mammalian NMJ forms as a result of mutually stimulating signaling from both MNs and skeletal muscle fibers. Neurons can provoke the post-synaptic terminal site at the muscle, and likewise, skeletal fibers can induce pre-synaptic differentiation of neurons^27^. One outcome is the clustering of AChRs, which are uniformly distributed throughout myotubes but become greatly concentrated at the post-synaptic membrane, due to both AChR redistribution throughout the membrane and increased synthesis^34^. Another outcome is the extension and branching of the neuron’s axon into a motor nerve terminal that can release neurotransmitters (such as ACh) at the NMJ. We observed both outcomes, signifying functional NMJ formation. The extension of neurites across 2D surfaces (Figure 3) was a promising observation, indicating the potential to extend neurites throughout engineered tissues and innervate the skeletal muscle. Indeed, we observed a similar phenomenon in 3D multi-layered tissue rings (Figure 6b).

In a functioning NMJ, muscle contraction is induced by an excitatory neurotransmitter that is released from an MN at the synaptic cleft between cells, binds to a post-synaptic receptor, and depolarizes the cell on which it acts, thus increasing that cell’s excitability and probability of firing an action potential^35^. When the nicotinic neurotransmitter ACh binds to its specific membrane receptor (AChR) on the muscle cell, it initiates an intracellular signaling cascade resulting in the release of calcium ions from the sarcoplasmic reticulum in the muscle fiber, terminating in actin–myosin contraction^10,36^. Before ACh is released, however, the MN must be chemically stimulated by an excitatory neurotransmitter that induces a neuronal action potential^35,37^. Various studies have reported the use of glutamate in chemical activation of neuromuscular systems with high success, as it is a major excitatory neurotransmitter in the mammalian nervous system. We demonstrate that the site-specific innervation of a group of muscle fibers in the multi-layered tissue rings allowed for muscle contraction via chemical stimulation of MNs, with the frequency of contraction increasing with glutamate concentration. The decrease in displacement per contraction followed a physiological relationship between force output and frequency for functional skeletal muscle^3,38^; the engineered tissue ring had less time to return to baseline tension between each successive stimulus as the frequency of neuronal firing increased. Because the addition of curare terminated the contractions, we confirmed both that the muscle contraction was MN-induced and the presence of a functional NMJ.

Further enhancements to the multi-layered tissue ring system could allow for the development of an autonomous biological actuator. With a bio-fabricated system permitting controllable mechanical and geometric attributes on a range of length scales, our novel engineered cellular system can be utilized for easier integration of other modular “building blocks” in living cellular and biological machines. This modular NMJ platform is the foundation of a novel heterotypic cellular system and has the potential to address larger challenges in medicine and biology. Target applications could include microscale tissue fabrication for organ-on-a-chip mimics of neurodegenerative diseases or drug screening for neuromuscular diseases in an autonomous platform.

## References

[bib1] Kamm RD, Bashir R. Creating living cellular machines. Annals of Biomedical Engineering 2013; 42: 445–459. 24006130 10.1007/s10439-013-0902-7 PMC4307756

[bib2] Raman R, Cvetkovic C, Uzel SGM et al. Optogenetic skeletal muscle-powered adaptive biological machines. Proceedings of the National Academy of Sciences of the United States of America of the United States of America 2016; 113: 3497–3502. 10.1073/pnas.1516139113 PMC4822586 26976577

[bib3] Cvetkovic C, Raman R, Chan V et al. Three-dimensionally printed biological machines powered by skeletal muscle. Proceedings of the National Academy of Sciences USA of the United States of America 2014; 111: 10125–10130. 10.1073/pnas.1401577111 PMC4104884 24982152

[bib4] Duffy RM, Feinberg AW. Engineered skeletal muscle tissue for soft robotics: Fabrication strategies, current applications, and future challenges. WIREs Nanomedicine and Nanobiotechnology 2014; 6: 178–195. 24319010 10.1002/wnan.1254

[bib5] Chan V, Park K, Collens MB et al. Development of miniaturized walking biological machines. Scientific Reports 2012; 2: 1–8. 10.1038/srep00857 PMC3498929 23155480

[bib6] Nawroth JC, Lee H, Feinberg AW et al. A tissue-engineered jellyfish with biomimetic propulsion. Nature Biotechnology 2012; 30: 792–797. 10.1038/nbt.2269 PMC4026938 22820316

[bib7] Williams BJ, Anand SV, Rajagopalan J et al. A self-propelled biohybrid swimmer at low Reynolds number. Nature Communications 2014; 5: 3081. 10.1038/ncomms4081 24435099

[bib8] Hinds S, Bian W, Dennis RG et al. The role of extracellular matrix composition in structure and function of bioengineered skeletal muscle. Biomaterials 2011; 32: 3575–3583. 21324402 10.1016/j.biomaterials.2011.01.062 PMC3057410

[bib9] Sakar MS, Neal D, Boudou T et al. Formation and optogenetic control of engineered 3D skeletal muscle bioactuators. Lab on a Chip 2012; 12: 4976–4985. 22976544 10.1039/c2lc40338b PMC3586563

[bib10] Marieb EN. Essentials of Human Anatomy and Physiology. Pearson/Benjamin Cummings: San Francisco, CA, USA; 2006: 279–326.

[bib11] Mars T, Yu KJ, Tang X-M et al. Differentiation of glial cells and motor neurons during the formation of neuromuscular junctions in cocultures of rat spinal cord explant and human muscle. Journal of Comparative Neurology 2001; 251: 239–251. 10.1002/cne.1312 11536191

[bib12] Larkin LM, van der Meulen JH, Dennis RG et al. Functional evaluation of nerve-skeletal muscle constructs engineered *in vitro*. In Vitro Cellular & Developmental Biology—Animal 2006; 42: 75–82. 16759152 10.1290/0509064.1

[bib13] Wichterle H, Lieberam I, Porter JA et al. Directed differentiation of embryonic stem cells into motor neurons. Cell 2002; 110: 385–397. 12176325 10.1016/s0092-8674(02)00835-8

[bib14] Miles GB, Yohn DC, Wichterle H et al. Functional properties of motoneurons derived from mouse embryonic stem cells. Journal of Neuroscience 2004; 24: 7848–7858. 15356197 10.1523/JNEUROSCI.1972-04.2004 PMC6729934

[bib15] Wichterle H, Peljto M. Current Protocols in Stem Cell Biology. John Wiley & Sons, Inc.: Hoboken, NJ, USA; 2008: 5 1H.1.1–1H.1.9. 10.1002/9780470151808.sc01h01s5 18770634

[bib16] Umbach JA, Adams KL, Gundersen CB et al. Functional neuromuscular junctions formed by embryonic stem cell-derived motor neurons. PLoS ONE 2012; 7: e36049. 22574134 10.1371/journal.pone.0036049 PMC3344836

[bib17] Dhara SK, Stice SL. Neural differentiation of human embryonic stem cells. Journal of Cellular Biochemistry 2008; 105: 633–640. 18759328 10.1002/jcb.21891 PMC2574851

[bib18] Guo X, Das M, Rumsey J et al. Neuromuscular junction formation between human stem-cell-derived motoneurons and rat skeletal muscle in a defined system. Tissue Engineering Part C 2010; 16: 1347–1355. 10.1089/ten.tec.2010.0040 PMC2988647 20337513

[bib19] Guo X, Gonzalez M, Stancescu M et al. Neuromuscular junction formation between human stem cell-derived motoneurons and human skeletal muscle in a defined system. Biomaterials 2011; 32: 9602–9611. 21944471 10.1016/j.biomaterials.2011.09.014 PMC3200565

[bib20] Kubo T, Randolph Ma, Gröger A et al. Embryonic stem cell-derived motor neurons form neuromuscular junctions *in vitro* and enhance motor functional recovery *in vivo*. Plastic and Reconstructive Surgery 2009; 123: 139S–148SS. 19182673 10.1097/PRS.0b013e3181923d07

[bib21] Larkin LM, Der V, Meulen JH et al. Functional evaluation of nerve-skeletal muscle constructs engineered *in vitro*. In Vitro Cellular & Developmental Biology—Animal 2006; 42: 75–82. 16759152 10.1290/0509064.1

[bib22] Uzel SGM, Platt RJ, Subramanian V et al. Microfluidic device for the formation of optically excitable, three-dimensional, compartmentalized motor units. Science Advances 2016; 2: e1501429. 27493991 10.1126/sciadv.1501429 PMC4972469

[bib23] Morimoto Y, Kato-Negishi M, Onoe H et al. Three-dimensional neuron-muscle constructs with neuromuscular junctions. Biomaterials 2013; 34: 9413–9419. 24041425 10.1016/j.biomaterials.2013.08.062

[bib24] Jacobs PF. Rapid Prototyping and Manufacturing: Fundamentals of StereoLithography. 1st edn. Society of Manufacturing Engineers: Dearborn, MI, USA; 1992.

[bib25] Melchels FPW, Feijen J, Grijpma DW. A review on stereolithography and its applications in biomedical engineering. Biomaterials 2010; 31: 6121–6130. 20478613 10.1016/j.biomaterials.2010.04.050

[bib26] E Michalska A. Isolation and propagation of mouse embryonic fibroblasts and preparation of mouse embryonic feeder layer cells. Current Protocols in Stem Cell Biology 2007; Chapter 1, Unit1C.3. 10.1002/9780470151808.sc01c03s3 18785164

[bib27] Meier T, Wallace BG. Formation of the neuromuscular junction: Molecules and mechanisms. BioEssays 1998; 20: 819–829. 9819569 10.1002/(SICI)1521-1878(199810)20:10<819::AID-BIES7>3.0.CO;2-N

[bib28] Vandenburgh HH, Karlisch P, Shansky J et al. Insulin and IGF-I induce pronounced hypertrophy of skeletal myofibers in tissue culture. American Journal of Physiology—Cell Physiology 1991; 260: C475–C484. 10.1152/ajpcell.1991.260.3.C475 2003574

[bib29] Duan C, Ren H, Gao S. Insulin-like growth factors (IGFs), IGF receptors, and IGF-binding proteins: Roles in skeletal muscle growth and differentiation. General and Comparative Endocrinology 2010; 167: 344–351. 20403355 10.1016/j.ygcen.2010.04.009

[bib30] Powell CA, Smiley BL, Mills J et al. Mechanical stimulation improves tissue-engineered human skeletal muscle. American Journal of Physiology—Cell Physiology 2002; 283: C1557–C1565. 12372817 10.1152/ajpcell.00595.2001

[bib31] Goldspink G, Scutt A, Loughna PT et al. Gene expression in skeletal muscle in response to stretch and force generation. American Journal of Physiology 1992; 262: R356–R363. 1373039 10.1152/ajpregu.1992.262.3.R356

[bib32] Chan V, Zorlutuna P, Jeong JH et al. Three-dimensional photopatterning of hydrogels using stereolithography for long-term cell encapsulation. Lab on a Chip 2010; 10: 2062–2070. 20603661 10.1039/c004285d

[bib33] Arcaute K, Mann BK, Wicker RB. Practical use of hydrogels in stereolithography for tissue engineering applications. In: Bártolo PJ, editor. Stereolithography: Materials, Processes and Applications. Springer: Boston, MA, USA; 2011: 299–331.

[bib34] Sanes JR, Lichtman JW. Maturation and maintenance of a postsynaptic apparatus. Nature Reviews Neuroscience 2001; 2: 791–805. 11715056 10.1038/35097557

[bib35] Koeppen BM, Stanton BA, editors. Berne & Levy Physiology. 6th edn. Mosby Elsevier: Philadelphia, PA, USA; 2008.

[bib36] Watras JM. Muscle. In: Koeppen BM, Stanton BA, editors. Berne & Levy Physiology. Mosby Elsevier: Philadelphia, PA, USA; 2008: 233–267.

[bib37] Campbell NA, Reece JB. Biology. 6th edn. Pearson Education, Inc.: San Francisco, CA, USA; 2002.

[bib38] King AM, Loiselle DS, Kohl P. Force generation for locomotion of vertebrates: Skeletal muscle overview. IEEE Journal of Oceanic Engineering 2004; 29: 684–691.

