# Supplementary material for: A 3D-printed platform for modular neuromuscular motor units
Source: Microsyst Nanoeng. 2017 Jun 19;3:17015. doi: 10.1038/micronano.2017.15 (PMC6444989; doi:10.1038/micronano.2017.15)
Supplement: Supplementary Information [file micronano201715-s1.pdf]

Supplementary file

A 3D printed platform for modular neuromuscular motor units

Caroline Cvetkovic[1,2], Max H. Rich[3], Ritu Raman[2,4], Hyunjoon Kong[3] and Rashid Bashir[1,2]

Microsystems & Nanoengineering (2017) **3,** 17015; doi:10.1038/micronano.2017.15; Published online: 19 June 2017

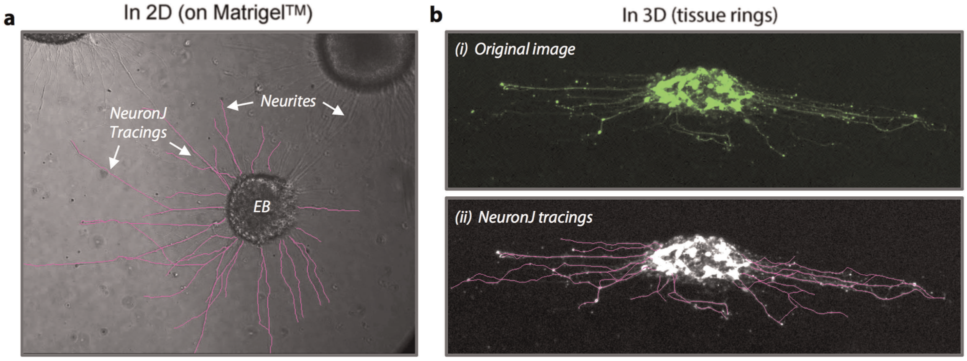

Figure S1 Examples demonstrating the use of NeuronJ, an ImageJ plugin, to measure the extension of neurites in (**a**) 2D Matrigel and (**b**) 3D tissue rings (image reproduced from Figure 6bi).

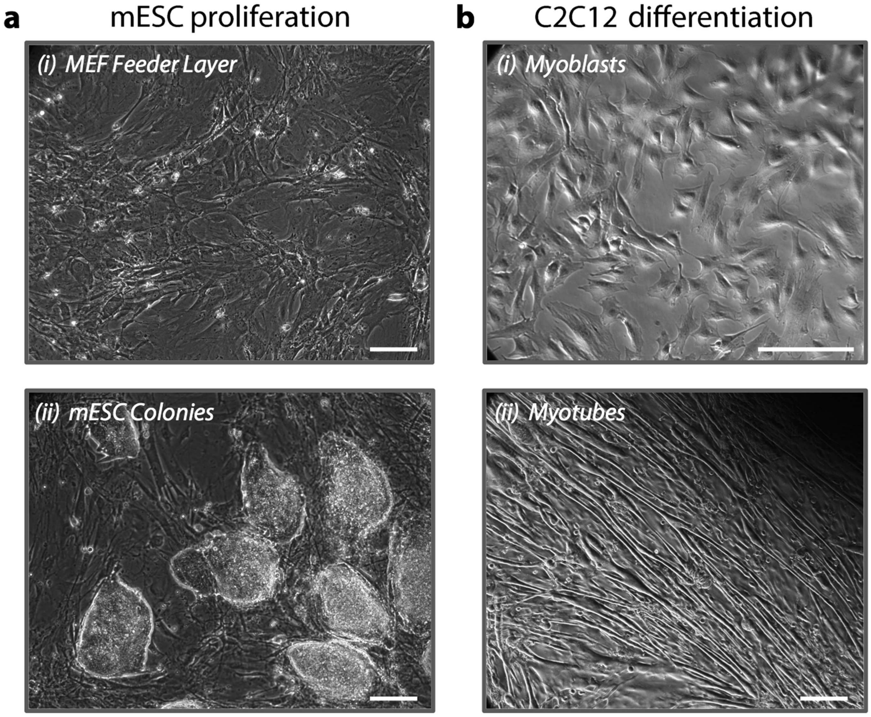

Figure S2 (**a**) (i) Mouse embryonic fibroblasts (MEFs) were seeded as a feeder layer prior to culture of (ii) mouse embryonic stem cells (HBG3 mESCs), which grew in colonies during proliferation. (**b**) During differentiation, C2C12 (i) myoblasts proliferated and then fused to form (ii) myotubes containing multiple nuclei. All scale bars, 200 μm.

[1]Department of Bioengineering, University of Illinois at Urbana-Champaign, Urbana, IL 61801, USA; [2]Micro and Nanotechnology Laboratory, University of Illinois at Urbana-Champaign, Urbana, IL 61801, USA; [3]Department of Chemical and Biomolecular Engineering, University of Illinois at Urbana-Champaign, Urbana, IL 61801, USA and [4]Department of Mechanical Science and Engineering, University of Illinois at Urbana-Champaign, Urbana, IL 61801, USA
Correspondence: Rashid Bashir (rbashir@illinois.edu)

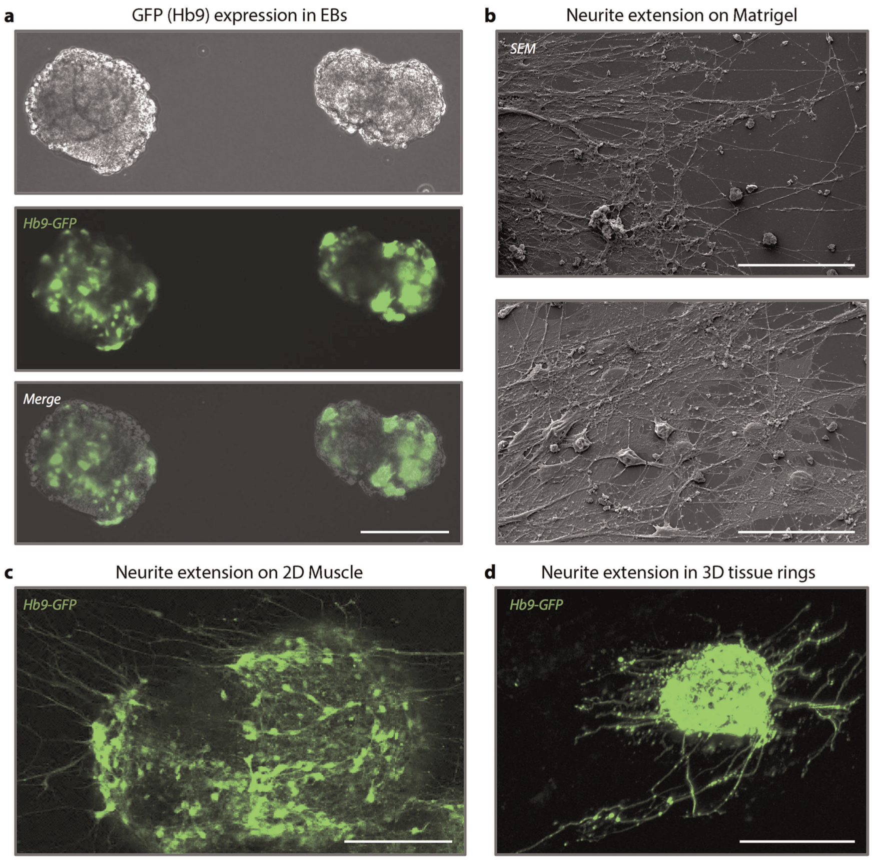

Figure S3 (**a**) EBs in suspension culture expressed GFP under the Hb9 promoter, providing a visual marker for motor neuron differentiation. Scale bars, 200 µm. (**b**) When allowed to adhere to an ECM-coated surface (here, on Matrigel), EBs extended neurites radially outward. These neurites formed synapses with each other, which could be visually observed with SEM imaging, as shown. Scale bars, 50 µm. (**c**) As on ECM substrates, EBs on C2C12s extended neurites outward, toward the muscle fibers. (**d**) A similar phenomenon was observed in 3D tissue rings. Scale bars, 200 µm.

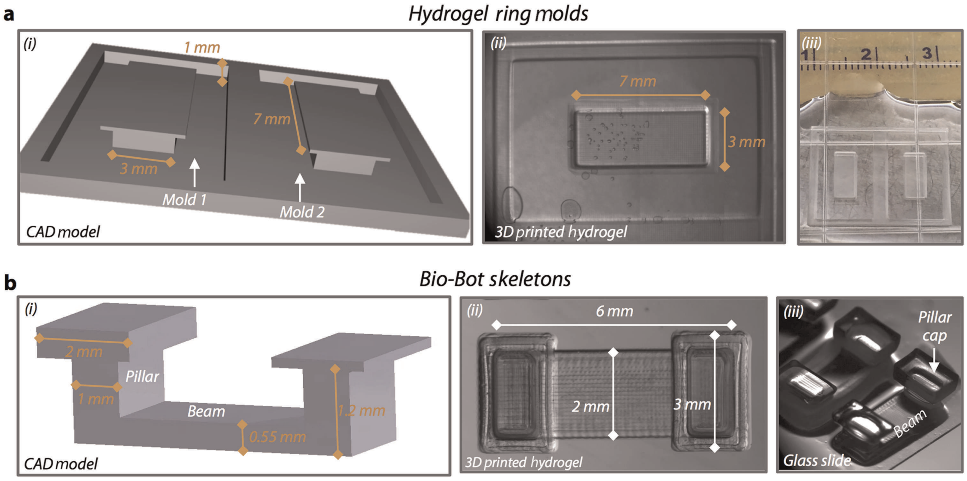

Figure S4 Designed and fabricated dimensions of (**a**) hydrogel ring molds and (**b**) bio-bot skeletons. Shown for both are (i) computer-aided design models, and (ii-iii) actual 3D printed hydrogels.

Supplementary Video 1 Confocal *z*-stacks demonstrating the presence of EBs and differentiated muscle (stained for MF20 myosin heavy chain) in two multi-layered tissue rings, 2 days after co-culture. Scale bars, 100 µm.

Supplementary Video 2 Spontaneous contraction was observed in mature muscle fibers in layer 1, both 2 and 24 h after adding layer 2 with EBs and beginning co-culture. Scale bars, 0.5 mm.

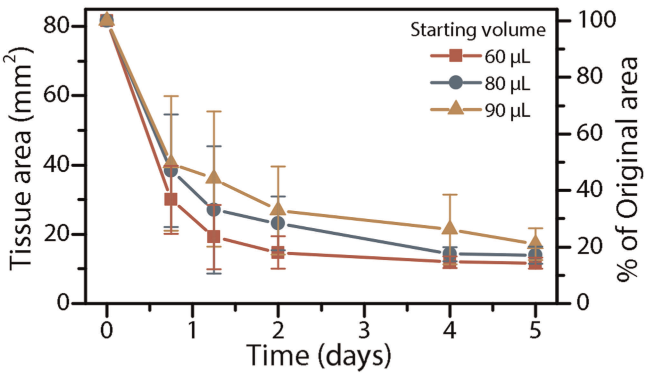

Figure S5 The thickness of the tissue was controlled by varying the initial cell-gel volume; initial volumes of 60, 80, and 90 µL (containing 3×10^5, 4×10^5, and 4.5×10^5 cells in each ring) resulted in ring tissues with layer 1 cross-sectional areas of 11.7 ± 1.6, 13.9 ± 2.4, and 17.1 ± 4.6 mm^2 (14.3, 17.1, and 21.0% of the original area) after 5 days, respectively. Plot represents mean ± s.d. ($n=3$–6 rings analyzed per time point and initial volume).

Supplementary Video 3 Confocal *z*-stack demonstrating the outward growth of neurites from GFP^+ EBs in a multi-layered tissue ring, 3 days after co-culture. Scale bars, 100 µm.

Supplementary Video 4 Muscle contraction was observed after chemical stimulation of MNs (via a bath application of glutamate), with the frequency of twitching increasing with glutamate concentration. The addition of 25 µM curare halted the contractions. Video recordings were begun 2 min after adding 200 and 400 µM glutamate, and 10 s after adding 25 µM curare. Arrows represent regions of maximum contraction. Scale bars, 1 mm.
